# Wissensmanagement in virtuellen Welten: Wissensgenerierung in Virtual Reality-Umgebungen

**DOI:** 10.1365/s40702-021-00814-z

**Published:** 2021-12-09

**Authors:** Johannes Schenk, Johannes Kurik, Johanna Gelberg, Andreas Lischka

**Affiliations:** Institute of Management & Information Systems (mis), FOM Hochschule für Oekonomie & Management, Leimkugelstraße 6, 45141 Essen, Deutschland

**Keywords:** Design Thinking, Kollaboration, Praxisbeispiel, Virtual Reality, Wissensgenerierung, Wissensmanagement, Design Thinking, Collaboration, Virtual Reality, Practical Example, Knowledge Creation, Knowledge Management

## Abstract

In Zeiten einer globalen Pandemie werden physische Meetings seltener. Eine beschleunigte Digitalisierung der Meeting-Modalitäten findet statt. Formelle und informelle Kommunikation läuft weitgehend virtuell ab, vor allem in den dienstleistungsorientierten Branchen der Wissensarbeit. Gerade die Wissensgenerierung in kreativen Prozessen ist ohne physische Präsenz eine Herausforderung. Das Generieren von innovativen Lösungen oder Produktideen erfordert Vertrauen und physische Nähe. Unsere Forschungsfrage lautet daher, inwiefern Virtual-Reality-Umgebungen im Vergleich zu Online-Meetings eine Verbesserung in Wissensschaffungsprozessen bieten können. Daher vergleichen wir Design Thinking Workshops in Virtual Reality mit konventionellen Kollaborationswerkzeugen, um mögliche Vorteile und weitere Anwendungsszenarien für die Wissensgenerierung in Virtual Reality zu identifizieren. Dabei steht die Messung der quantitativen Wirkung von Virtualität auf Faktoren wie Teamzusammenhalt, Kreativität, Kommunikation und Ideengenerierung im Vordergrund. Darüber hinaus analysieren wir qualitative Aspekte wie Verbesserungspotenziale und mögliche bevorzugte Anwendungsfälle für die unterschiedlichen Formen der virtuellen Zusammenarbeit.

## Abstract (en)

In times of a global pandemic physical meetings become rare. An accelerated digitalization of meeting modalities takes place. Formal and informal communication largely takes place virtually, especially in service-orientated knowledge work industries. Especially knowledge generation in creative processes is challenging without physical presence. Inventing innovative solutions or product ideas requires trust and physical closeness. Therefore, our research question is to what extent virtual reality environments can offer an improvement in knowledge creation processes compared to online meetings. Hence, we compare Design Thinking workshops in VR with conventional collaboration tools, to identify potential benefits and further application scenarios for knowledge creation in VR. Thereby, the measurement of the quantitative effect of virtuality on factors like team-cohesion, creativity, communication and idea generation is the center of attention. Furthermore, we analyze qualitative aspects like potential for improvement and possible preferred use cases for each form of virtual collaboration.

## Einführung

Die Zusammenarbeit in räumlich verteilten Teams führt nicht nur zu neuer Flexibilität, sondern auch zu Herausforderungen aufgrund der räumlichen Distanz. Insbesondere in den Bereichen Kommunikation, Transparenz, dem notwendigen Wissenstransfer und der Entwicklung gemeinsamer Wissensräume bzw. Netzwerke warten ungeahnte Herausforderungen. Virtuelle Teams sind langsam und fehleranfällig, insbesondere beim Informations- und Wissensaustausch (Olson et al. 2008; Rosen et al. 2007). Eine Reihe spezifischer Herausforderungen, wie soziale und kulturelle Distanz, die Lokalisierung relevanter Wissensquellen, Missverständnisse in der Kommunikation sowie fehlende formale Regeln und Reflexionsmöglichkeiten führen zu der Frage, was insbesondere kleine und mittlere Unternehmen (KMU) im Wissensmanagement in Bezug auf virtuelle Zusammenarbeit verbessern können. Die Covid-19-Pandemie verstärkt viele dieser Herausforderungen durch soziale Distanzierung, Home-Office und verstärkte virtuelle Teamarbeit und Kollaboration zusätzlich. In den vergangenen Pandemie-Monaten mussten sich vor allem KMUs diesen Herausforderungen stellen, da sie in der Digitalisierung ihrer Arbeitsprozesse oft nicht so weit fortgeschritten sind wie internationale Unternehmen oder Großkonzerne. Durch die pandemiebedingte Verschiebung von klassischen Arbeitsmodellen hin zum vermehrten Einsatz von mobilen und hybriden Arbeitsformen wurde die Notwendigkeit einer Neuerfindung des virtuellen Arbeitens sowohl für die Organisationen als auch für die Wirtschaftsinformatik deutlich.

Virtuelle Meetings finden heutzutage auf verschiedenen virtuellen Plattformen und technisch geeigneten Lösungen statt. Wichtigste Voraussetzung ist die Möglichkeit des Austauschs über verschiedene Medien, wie Text, Ton oder Bild sowie Kollaborationsfeatures wie Screen-Sharing oder geteilte Listen bzw. Whiteboards. Bereits etablierte Videokonferenzsysteme wie Cisco Webex oder Zoom sowie Kollaborationstools wie MS Teams oder Slack haben ihren Weg in die Arbeitswelt gefunden (Balogh und Dinu 2021; Litsa 2017). Sie decken die notwendige formelle Kommunikation ab, indem sie digitale Konferenzen und Zusammenarbeit ermöglichen (Panopto 2019). Informelle Faktoren, wie Körpersprache, der Aufmerksamkeitsfokus oder die Tatsache, ob sich die Teilnehmenden bereits vorab virtuell oder real kennen lernen konnten, bleiben jedoch eher unbeachtet (Kessler 2020). Die Möglichkeiten dieser Tools in kreativen Prozessen wie der Entwicklung neuer Ideen sind begrenzt, da die gemeinsame physische Präsenz fehlt. Darüber hinaus fehlt es virtuellen Teams in Videokonferenzen an Teamzusammenhalt und Vertrauen (Mueller et al. 2011) aufgrund der fehlenden nonverbalen Kommunikation. Eine mögliche Lösung, diesen Herausforderungen entgegenzuwirken, könnte der Einsatz von Virtual Reality (VR) für virtuelle Meetings sein, um eine immersive und vertrauenswürdige Umgebung zu schaffen (Mueller et al. 2011). Dies scheint insbesondere in den Bereichen der Wissensvermittlung (Wallet et al. 2008) und Remote Kollaboration (Piumsomboon et al. 2017) aussichtsreich. Das Management-Paradigma eines wissensschaffenden Unternehmens erklärt, wie der stark zunehmende Einsatz von Informationstechnologie Unternehmen helfen kann Effizienzvorteile in einer entstehenden Wissensgesellschaft zu realisieren (Liao 2003; Nonaka et al. 1996). Die zentrale Frage dieses Artikels lautet daher, ob und wie VR-Technologie, bei der die Teilnehmer in eine simulierte Umgebung eintauchen (Milgram und Kishino 1994) und über ihre Avatare realitätsnah interagieren (Mueller et al. 2011), den Herausforderungen bezüglich Wissensmanagement in virtuellen Teams entgegenwirken kann. Dabei werden die Vor- und Nachteile sowie die Einsatzgebiete der VR-Technologie im Vergleich zu herkömmlichen Kollaborationswerkzeugen betrachtet. Darüber hinaus zielen wir darauf ab, spezifische Einsatzgebiete zu identifizieren, in denen der Einsatz von VR klar im Vorteil ist, um damit virtuelle Teams zum wertschöpfenden Einsatz der Technologie in Wissensgenerierungsprozessen zu befähigen. Für eine effektive Nutzung und Integration von VR in virtuelle Arbeitsabläufe scheint eine spezifische methodische und organisatorische Unterstützung notwendig. Alles in allem, untersuchen wir den Einsatz und die Auswirkungen der VR-Technologie bei der Wissensgenerierung in virtuellen Teams im Gegensatz zu herkömmlichen Online-Kollaborationstools.

## Konzeptuelle Grundlagen

### Virtual Reality als aufstrebende Technologie

Neu aufstrebende Technologien führen üblicherweise zur sorgfältigen Evaluierung möglicher Anwendungs- und Einsatzszenarien, sobald sie marktreif werden. Für VR geschah dies 2017, als sie auf Gartners „Pfad der Erleuchtung“ nach oben kletterte (Panetta 2017). Dennoch verschwand VR in der zweiten Hälfte des Jahres 2018 überraschend aus dem Hype-Zyklus (Panetta 2018). Seltsamerweise hat VR nie das sogenannte „Plateau der Produktivität“ erreicht. Der Grund dafür ist, dass Gartner die Technologie als bereits ausgereift (bzw. marktreif) ansah und daher VR nicht mehr als neue Technologie bewerten konnte (Bastian 2018).

Bereits Mitte der 1960er-Jahre wurden die konzeptionellen Grundlagen für VR gelegt (Sutherland 1965). Ein weiterer Meilenstein ist der Beginn der Vermarktung durch die Videospielindustrie Mitte der 1990er-Jahre mit Nintendos Virtual Boy VR-Headset (Edwards 2020). Die Anerkennung von VR in der Wissenschaft und Forschung steigt (Wohlgenannt et al. 2020), während die Technologie in neue Märkte, die Breite der Gesellschaft sowie die produktive Arbeitswelt vordringt.

VR ist heute eine marktreife Technologie mit exponentiell steigendem Kundeninteresse und prognostizierten steigenden Marktanteilen (Bitkom 2020; IDC 2021; SuperData Research 2020). Mit dem Kauf von Oculus durch Facebook im Jahr 2014 (Solomon 2014) gibt es einen Big Player, der in hohem Maße in eine neue Medienplattform investiert, mit fast 10.000 Mitarbeitern, die im Jahr 2020 in einem einzigen Unternehmen an VR-Technologie arbeiten (Heath 2021). Analysten erwarten, dass diese Technologie mit bestehenden Märkte wie z. B. für PCs oder Smartphones konkurrieren wird (Bellini et al. 2016). Daher sind Unternehmen aufgefordert, den möglichen Einsatz von VR in ihren Prozessen zu evaluieren, da die Technologie voranschreitet und weltweit führende Unternehmen stark in sie investieren (Harvard Business Review 2019). Neben den Hauptanwendungsfeldern Gaming und Wissensvermittlung wird VR insbesondere im Medizin- und Gesundheitswesen, Sport, Architektur- und Immobilienbereich und sogar im Tourismus eingesetzt (Klöß 2021; Wohlgenannt et al. 2020).

Technologisch umgesetzt wird VR durch den Einsatz von sogenannten „Head-Mounted Displays“ (kurz: HMD) wie z. B. Computer-Displays in oder auf einer Brille definiert, die in der Lage sind, eine umfassende Illusion von Realität zu vermitteln (Slater und Wilbur 1997). Die Hauptaspekte von VR umfassen aus wissenschaftlicher Sicht insbesondere *Immersion, Präsenz* und *Interaktivität* (Ryan 2001; Radianti et al. 2020; Walsh und Pawlowski 2002), mithilfe derer stärkere Ausprägungen von VR erreicht werden können als mit anderen Informationssystemen (Mütterlein und Hess 2017).

*Immersion* beschreibt dabei die Verbindung zwischen virtueller Welt und Nutzenden, wobei das Zeitgefühl und die reale Welt oft abgekoppelt werden. Der Fokus wird stattdessen auf die Aufgabenumgebung verlagert und die Sinne der Nutzenden werden von der realen Welt isoliert (McCall et al. 2004).

*Präsenz* ist die subjektive Erfahrung, an einem Ort oder in einer Umgebung zu sein, auch wenn man sich im übertragenen Sinne physisch an einem anderen Ort befindet (McCall et al. 2004).

*Interaktivität* ist ein Zustand, in dem „ein Benutzer die VR-Umgebung in Echtzeit verändern kann“ (Radianti et al. 2020) bzw. mit ihr interagieren kann. Ein Beispiel dafür ist das Drücken eines Schalters, welcher das Öffnen einer Tür bewirkt.

### Entwicklung der Wissensgenerierung als Wissensmanagementmethode

**Abb. 1:**
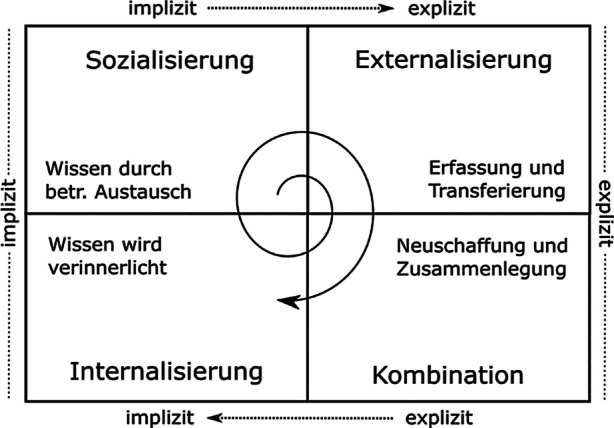
SECI-Modell nach Nonaka und Takeuchi (1995)

Unsere Forschung baut auf der Konzeptualisierung der Wissensgenerierung auf, wie sie von Nonaka und Takeuchi vorgeschlagen wurde (Nonaka und Takeuchi 1995). Nonaka spezifiziert vier Wissensgenerierungsmodi als Prozesse des Zusammenspiels zwischen implizitem und explizitem Wissen, die zur Schaffung von neuem Wissen führen. Implizites Wissen ist dabei personengebundenes, intuitives Wissen (wie z. B. Fahrrad fahren), welches eigene Erfahrungen und „Learning-by-Doing“ erfordert, während explizites Wissen formales und dokumentierbares Wissen (wie z. B. Aufpumpen eines Fahrradreifens) darstellt, welches damit leichter erklärbar ist. Gemäß Nonaka lässt sich der Prozess der Wissensgenerierung in die unterschiedlichen Phasen *Sozialisierung, Externalisierung, Kombination* sowie *Internalisierung* einteilen. Diese Konzeptualisierung wird mit dem Akronym SECI bezeichnet. Vereinfacht gesagt, geschieht die Transformation von implizitem in explizites Wissen in den Phasen *Sozialisierung* und *Externalisierung*, während die Rückführung von explizitem in implizites Wissen in den Phasen *Kombination* und *Internalisierung* stattfindet (siehe Abb. 1).

*Sozialisierung* stellt dabei den Erwerb von Wissen in der betrieblichen bzw. organisationalen Zusammenarbeit dar. Dabei entsteht neues implizites Wissen, das durch informelle Interaktion aufgebaut wird (z. B. bei alltäglicher Zusammenarbeit).

*Externalisierung* ist die Umwandlung von impliziten Wissen in explizites Wissen durch Dokumentation und Erfahrungsaustausch (z. B. Experteninterviews).

*Kombination* bezieht sich auf die Zusammenlegung von zuvor nicht miteinander verbundenen Wissensdomänen und beinhaltet das Sammeln, Verarbeiten und Sortieren von vorhandenem explizitem Wissen (z. B. durch Diskussionen).

*Internalisierung* ist die Verinnerlichung des expliziten, von Organisationen absorbierten Wissens, in individuell gehaltenes und organisational verankertes implizites Wissen (z. B. Erfahrungen aus Experimenten (Kale und Singh 1999)).

**Abb. 2:**
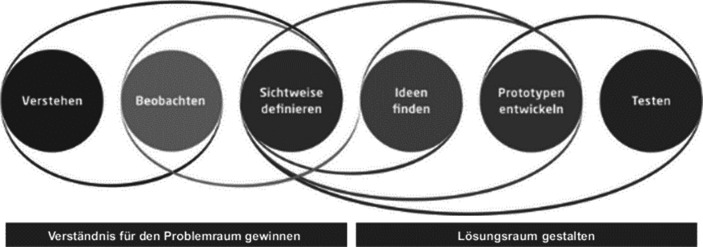
Design Thinking (Hasso-Plattner-Institut 2021)

Darüber hinaus haben wir Design Thinking (DT) als geeignete praktische Methodik für Wissensgenerierung für unsere Untersuchungen gewählt, da es einen anerkannten, kontrollierten Wissenserstellungsprozess darstellt. DT bietet großes Potenzial für prototypische Anwendungsforschung und hat sich bereits in bestehender Wirtschaftsinformatik-Forschung etabliert (Vogel et al. 2021, 2020; Fromm et al. 2021). DT ist ein Ansatz zur kreativen Problemlösung, der sich besonders für interdisziplinäre Teams eignet, um innovative und nutzerzentrierte Produkte und Dienstleistungen zu entwickeln. Darüber hinaus stellt DT eine Schlüsselmethodik zur Verbesserung der Kreativität dar, während es sich zu einer höchst relevanten Arbeitsmethode entwickelt (Accenture 2019). Globale Konzerne wie Google oder SAP nutzen die Methode der nutzerzentrierten Wissensgenerierung, um disruptive Innovationen zu erfinden. DT umfasst die folgenden iterativen Schritte im Wissensgenerierungsprozess: *Verstehen, Beobachtung, Sichtweisen definieren, Ideen finden, Prototypen entwickeln* sowie *Testen* (siehe Abb. 2).

In der bisherigen Forschung hat die DT-Phase *Prototypen entwickeln* eindeutig am meisten Aufmerksamkeit erhalten (Vogel et al. 2021, 2020; Fromm et al. 2021). Im Gegensatz dazu konzentriert sich unsere Forschung insbesondere auch auf die vorhergehenden Phasen. Die wissenschaftliche Lücke der sehr eingeschränkten Berücksichtigung der DT-Phasen in VR-Versuchssettings schließen wir mit einem erweiterten experimentellen Design unter Zuhilfenahme einer größeren Anzahl von miteinander in VR interagierenden Personen. Davon erhoffen wir uns auch Rückschlüsse auf die soziale Interaktion bei der Wissensgenerierung zwischen einzelnen Individuen und Teams in VR. Die Phasen bzw. Ziele von DT aus Abb. 2 finden sich dabei insbesondere im Versuchsaufbau der jeweiligen Experimente wieder (siehe 3.2). Die ersten drei Phasen haben dabei ein gemeinsames Problemraumverständnis zum Ziel, während die drei darauf anschließenden Phasen der Gestaltung des Lösungsraums dienen. Aufgrund unseres Interesses an der Wissensgenerierung in VR fokussieren wir beim DT-Ziel der Lösungsraumgestaltung insbesondere die DT-Phase *Ideen finden*.

## Forschungsdesign

### Forschungshypothesen

**Abb. 3:**
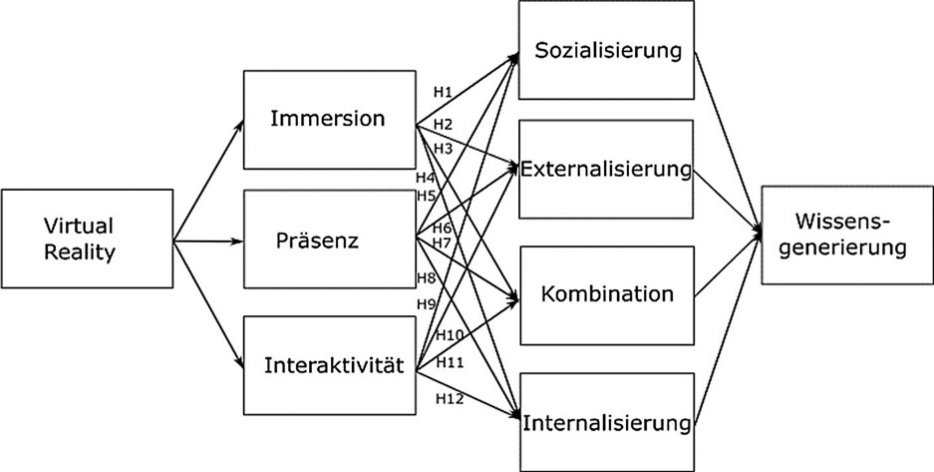
Forschungsmodell Wissensgenerierung in VR. (VR nach Ryan 2001; Radianti et al. 2020; Walsh und Pawlowski 2002. Wissensgenerierung nach Nonaka und Takeuchi 1995)

In der praktischen Anwendung befasst man sich in Hinblick auf VR hauptsächlich mit der Verbesserung der zugrundeliegenden Technologie, die inzwischen einen hohen Reifegrad und technischen Fortschritt erreicht hat (Klöß 2021). Die akademische Forschung hingegen beschäftigt sich darüber hinaus intensiv mit sozialen Faktoren wie kollaborativem Verhalten und körperbasierter Kommunikation in Wissensaustausch und -Vermittlung in VR (z. B. Fu et al. 2020). Aus dieser Diskrepanz ergibt sich unserer Einschätzung nach eine Forschungslücke bezüglich des Verständnisses von Potenzial, Nutzen und Mehrwert der Technologie zwischen praktischer Anwendung und theoretischer Forschung (Kneisel et al. 2019). Aus diesem Grund wollen wir mit einem realistischen Anwendungsszenario VR in Kollaborationsszenarien bzw. -umgebungen erproben und damit diese abweichenden Sichtweisen einander näherbringen. Unsere grundlegende Annahme ist, dass VR die Wissensgenerierung im Allgemeinen verstärken kann. Deshalb haben wir ein Forschungsmodell aufgestellt, das eine Brücke von den VR-Kerneigenschaften *Immersion, Präsenz* und *Interaktivität* (Ryan 2001; Walsh und Pawlowski 2002) zu den Wissensgenerierungsmodi *Sozialisierung, Externalisierung, Kombination, Internalisierung* – entsprechend dem weit verbreiteten SECI-Modell (Nonaka und Takeuchi 1995) schlägt. Abb. 3 veranschaulicht das Forschungsmodell und Tab. 1 zeigt die zugrunde liegenden Forschungshypothesen auf.

**Tab. 1:** Hypothesenmatrix

VR // SECI	Sozialisierung	Externalisierung	Kombination	Internalisierung
*Immersion*	H1 (+)	H2 (+)	H3 (+)	H4 (+)
*Präsenz*	H5 (+)	H6 (+)	H7 (+)	H8 (+)
*Interaktivität*	H9 (+)	H10 (+)	H11 (+)	H12 (+)

H1–H4 unterstellen dabei je einen positiven Einfluss von *Immersion*, H5–H8 entsprechend je einen positiven Einfluss von *Präsenz* und H9–H12 dementsprechend je einen positiven Einfluss von *Interaktivität* auf jeweils die vier SECI-Modi (*Sozialisierung, Externalisierung, Kombination* sowie *Internalisierung*). Ein Beispiel wie Abb. 3 in Verbindung mit Tab. 1 gelesen wird, lautet exemplarisch für die Hypothese H2: „Das Erleben bzw. Eintauchen in die virtuelle Umgebung (*Immersion*) hat einen positiven Einfluss auf die SECI-*Externalisierung*“. Eine detaillierte Übersicht mit ausformulierten Hypothesen findet sich in Tab. 5.

### Versuchsaufbau der Experimente

Um die potenziellen Vorteile von VR in Bezug auf die Wissensgenerierung zu untersuchen, vergleichen wir eine Fallstudie, die in VR durchgeführt wurde, mit einer gleichwertigen Fallstudie, die in einem herkömmlichen virtuellen Kollaborationstool durchgeführt wurde. Beide Fallstudien beinhalteten also vergleichbare Aufgaben zur Wissensgenerierung und basieren auf dem DT-Prozess.

Um unser Forschungsziel zu erreichen, mussten wir eine Vielzahl von Anforderungen (technische, organisationale, soziale etc.) berücksichtigen, während wir nach geeigneter und funktionsmäßig vergleichbarer Software suchten, um die Studie in beiden Umgebungen (VR und konventionell) durchzuführen. Daher bauten wir auf der Capability Map für festgelegten Anforderungen an Kompetenzen für virtuelle Zusammenarbeit (Lischka und Gelberg 2021) auf, um sowohl eine anpassbare VR-Umgebung als auch ein geeignetes herkömmliches Kollaborationswerkzeug auszuwählen. Darüber hinaus schlug uns einerseits eine hinzugezogene Technologie-Expertin drei potenzielle VR-Software-Tools (Spatial, MeetinVR und EngageVR) vor, die durch vier wissenschaftlich Mitarbeitende und Research Fellows in jeweils einer mindestens halbstündigen Session ausgiebig getestet wurden. Andererseits wurden reale Projektmeetings unter Verwendung der jeweiligen technischen Möglichkeiten und Features der entsprechenden VR-Tools durchgeführt. Diese Testungen fanden mithilfe von VR-Equipment und unter Verwendung eines vordefinierten Anforderungskatalogs statt. Dieser bestand aus 36 Kriterien, welche auf der Capability Map basieren und von den Testpersonen einzeln für jedes VR-Tool bewertet wurden. Zu den Kriterien gehörten Merkmale wie die Möglichkeiten zur Raumausstattung (z. B. Whiteboards und Haftnotizen), Übertragungsqualität, Benutzerfreundlichkeit, Multi-User-Möglichkeit sowie Aufzeichnungsmöglichkeiten, aber auch Faktoren wie Realitätsnähe oder Flow-Gefühl (Csikszentmihalyi 2014). Diese Ergebnisse dieser Bewertung ergaben, dass Spatial im Mittelwert 14,7 Kriterien erfüllte, MeetinVR 18,7 und EngageVR 23,3. Damit war die Entscheidung eindeutig zugunsten von EngageVR gefallen.

Andererseits mussten wir ein konventionelles Kollaborationswerkzeug auswählen, das einen vergleichbaren Funktionsumfang zu VR bietet. Dafür haben wir weitere Literaturergebnisse mit einbezogen (Tsai 2018) und als Resultat MS Teams als etabliertes Kollaborationstool insbesondere im KMU-Umfeld identifiziert. MS Teams hat weltweit 75 Mio. täglich aktive Nutzer (Spataro 2020) und ist damit eines der am weitesten verbreiteten Tools. Daher haben wir uns für ein vergleichendes Experiment zwischen MS Teams und EngageVR entschieden. Für eine bessere Abgrenzung beider Tools zeigt Tab. 2 eine Gegenüberstellung unterschiedlicher Kollaborationsfeatures auf.

**Tab. 2:** Gegenüberstellung unterschiedlicher Kollaborationsfeatures von MS Teams und EngageVR

Features // Tools	MS Teams	EngageVR
*Geteilte Whiteboards*	Tastatur und Freihand-Cursor	Tastatur und Freihand-Handheld
*Teilnehmerdarstellung*	Webcam mit Hintergrund	Eigens personalisierter Avatar
*Screen Sharing*	Desktop neben Webcams	Virtuelle(r) Bildschirm(e)
*Erweiterte Audiofeatures*	Break-Out Gruppenräume	3D Audio nach Entfernung
*Objektdarstellung*	2D Text- und Bildelemente	3D Texturelemente (IFX)
*Plattformfähigkeit*	Desktop, Mobile	Desktop, Mobile, VR-Plattformen

Die ausgewählte VR-Umgebung legt den Fokus auf Zusammenarbeit und Ideenfindung (Wissensgenerierung mithilfe von Kreativitätstechniken) und ist demzufolge ausgestattet. Dementsprechend bietet MS Teams ähnliche Features wie z. B. gemeinsame Dokumentenbearbeitung oder multi-user fähige Whiteboards und Listen. Um die Vergleichbarkeit noch weiter zu gewährleisten, haben beide Gruppen in unterschiedlicher Abfolge jeweils beide Fallstudien durchgeführt (randomisiertes Cross-Over Design), um mögliche Störeinflüsse z. B. durch die Reihenfolge der Versuchsdurchführung zu minimieren und die interne Validität zu erhöhen. Zur Bearbeitung der MST Fallstudie wurde eine Aufgabe in MS Teams gelöst, während bei der VR-Fallstudie Virtual-Reality-Headsets genutzt wurden, um eine entsprechende Aufgaben in EngageVR zu lösen. Beide Gruppen bestanden aus vier bis fünf Wirtschaftsinformatik-Studierenden, die bereits über umfangreiche Berufserfahrung verfügen und sich in der Durchführung der Versuchsaufgaben abwechselten. Selbstverständlich erhielten alle Versuchsteilnehmenden eine Einführung in die Nutzung beider Technologien.

**Abb. 4:**
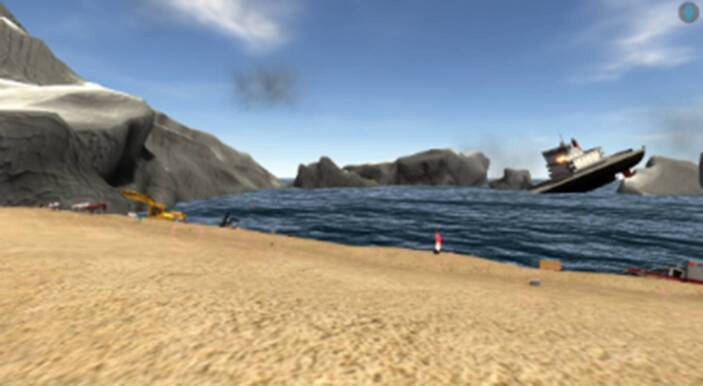
VR-Strandumgebung

**Abb. 5:**
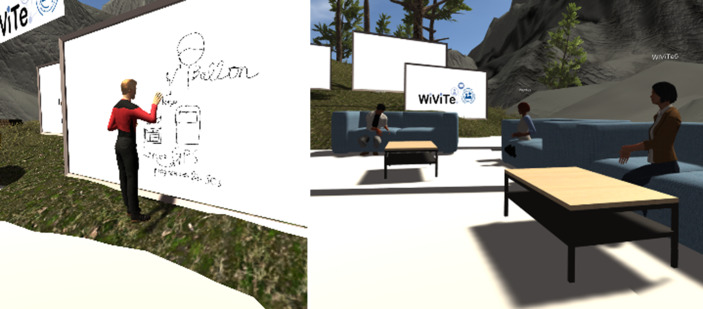
VR-Forschungslabor

Unsere VR-Fallstudie baut ein Narrativ auf, in dem die Teilnehmenden sich nach einem Schiffbruch am Strand einer verlassenen Insel wiederfinden (siehe Abb. 4). Nachdem sie das Treibgut erkundet und fünf von insgesamt 25 Gegenständen (z. B. Axt, Kamera, Luftballons) ausgewählt haben, die sie am Strand finden können, machen sie sich auf den Weg zu einem zurückgelassenen Forschungslabor (siehe Abb. 5), wo sie mithilfe dieser Gegenstände eine möglichst aussichtsreiche Rettungsmission planen.

Beide Umgebungen (Strand und Forschungslabor) haben wir an den DT-Prozess angelehnt, um die entsprechenden Ziele der DT-Phasen zu berücksichtigen. Die Strandumgebung diente zum Verständnis des Problemraums. Andererseits galt es im Forschungslabor entsprechende Lösungen zu gestalten. Außerdem zielten die beiden unterschiedlichen VR-Umgebungen auf die entsprechenden SECI-Phasen ab. Dabei standen am Strand die *Sozialisierung* und *Externalisierung* im Vordergrund, während im Forschungslabor *Kombination* und *Internalisierung *fokussiert wurden. Am Strand wurde so eine informelle Teambildungserfahrung geschaffen, wohingegen im Forschungslabor ein formeller Arbeitskontext simuliert wurde (siehe Tab. 3).

**Tab. 3:** Dimensionen beider VR-Umgebungen

Dimension // VR-Umgebung	Strandumgebung	Forschungslabor
*DT-Ziel*	Problemraumverständnis	Lösungsraumgestaltung
*SECI-Phasen*	*Sozialisierung* & *Externalisierung* (implizit → explizit)	*Kombination* & *Internalisierung* (explizit → implizit)
*Formalität*	Informell	Formell

Passend dazu, beinhaltete die MS Teams Fallstudie ein ähnliches Szenario. Im ersten Teil wurden die Teilnehmenden durch Textelemente in eine vergleichbares Narrativ eingeführt, in dem sie sich nach einer Not-Landung mit abgestürzter Landefähre auf dem Mond wiederfanden. Im zweiten Teil mussten sie entsprechende wichtige Gegenstände anhand von geteilten Listen priorisieren (z. B. Sauerstofftank, Seil, Revolver), um sich damit zu Fuß zu einer weit entfernten Mond-Basis zu retten. Genau wie beim VR-Versuchsaufbau wurden die jeweiligen DT-Ziele durch diese Aufteilung berücksichtigt. Die Durchführung einer thematisch identischen Versuchsdurchführung wäre zu bevorzugen gewesen. Dies ist allerdings aufgrund des Kreuz-Designs, in dem die gleichen Versuchspersonen jeweils beide Durchführungen versetzt und nacheinander durchlaufen, nicht möglich gewesen.

## Datenanalyse und Ergebnisse

Um die Ergebnisse der Fallstudiendurchführung zu analysieren und zu bewerten, haben wir die virtuellen Sitzungen jeweils aufgezeichnet. Darüber hinaus haben wir mit einem Online-Fragebogen quantitative und qualitative Daten der Teilnehmenden erhoben. Dieser Fragebogen stützt sich auf vorherige Studien, um die Anwendbarkeit und Vergleichbarkeit zu gewährleisten (Vogel et al. 2020; Mueller et al. 2011; Schulze und Hoegl 2006). Auf Basis dieser webbasierten Datenerhebung haben wir die Bewertungen der Teilnehmenden quantitativ ausgewertet.

Der Online Fragebogen umfasste demographische und berufliche Angaben (6 Fragen), Vorerfahrung bezüglich Online-Kollaborationstools und VR (6 Fragen), quantitative Forschungsfragen (72 Fragen in 6 Abschnitten, jeweils vergleichend zu VR und MS Teams) und abschließend offen gestellte qualitative Fragen (4 Fragen). Zur Auswertung in diesem Artikel wird hauptsächlich auf die Auswertung der quantitativen Forschungsfragen zurückgegriffen.

Die Versuchsgruppe ordnet sich zu einem Drittel dem weiblichen Geschlecht und zu zwei Drittel dem männlichen Geschlecht (*w* *=* *3, m* *=* *6, d* *=* *0*) zu. Die Mehrheit der Teilnehmenden ist zwischen 18 und 25 Jahren und vereinzelt zwischen 26 und 35 Jahren (*18–25 Jahre* *=* *7, 26–35 Jahre* *=* *2*). Der berufliche Hintergrund sowie die Teamgröße im Unternehmen sind angemessen heterogen verteilt. Die Versuchspersonen wurden aus dem Hochschul-Modul „Grundlagen der digitalen Transformation“ der Bachelor-Studiengänge „Business Administration“ sowie „Management & Digitalisierung“ rekrutiert. Als Dualstudierende in fortgeschrittenen Semestern verfügen die Teilnehmenden über mindestens zweijährige Berufserfahrung aus dem produzierenden Gewerbe oder der Dienstleistungsbranche. Daher weisen sie auch überwiegend Vorerfahrung in MS Teams auf, während Vorerfahrung mit VR nur in geringem Maße aus privaten Umfeld angegeben wurde. Die Versuchsdauer war im Vorfeld auf jeweils 30 min pro Durchführung (VR bzw. MS Teams) ausgelegt und wurde im Versuchsverlauf aufgrund technischer Verzögerungen auf 45 min angehoben.

Jede Hypothese wurden im Fragebogen mit sechs (je drei für VR und MS Teams) Frageitems abgedeckt und auf einer fünfstufigen Likert-Skala abgefragt (*1* *=* *„stimme überhaupt nicht zu“ bis 5* *=* *„stimme voll und ganz zu“*). Die Frageitems wiederum wurden entsprechend über die Errechnung des Mittelwertes zusammengefasst. Alle nachfolgenden statistischen Berechnungen wurden mit IBM SPSS Statistics Release 26.0.0.0 durchgeführt. Aufgrund der kleinen Stichprobengröße (*N* *=* *9*) wurde ein Shapiro-Wilk Test auf Normalverteilung der Stichproben durchgeführt. Dieser weist für alle Hypothesen außer H3 und H12 eine Normalverteilung der zugrundeliegenden Grundgesamtheit nach (*H3, H12: p*_*SW*_ *<* *0,05; H1–H2 und H4–H11: p*_*SW*_ *>* *0,05*). Folglich werden H3 und H12 nicht weiter berücksichtigt, da sich bei den entsprechenden Frageitems widersprüchliche Ausreißer in den dazugehörigen Antworten befinden. Diese sind möglicherweise auf Eingabefehler oder Verwechslungen seitens der Versuchsteilnehmenden zurückzuführen.

Daraufhin haben wir einen gepaarten t‑test für abhängige Stichproben durchgeführt, da die Werte für VR und MS Teams entsprechend von den gleichen Versuchspersonen erfasst wurden. Dieser dient zur Feststellung, ob die Mittelwerte der entsprechenden Stichproben verschieden sind und damit ein statistisch signifikanter Unterschied zwischen dem Stichprobenmittelwert beider abhängiger Gruppenpaare vorliegt. Dieser weist zumindest für H2 (*p*_*H2*_ *=* *0,02*) eine eindeutige statistische Signifikanz (p_t‑test_ < 0,05) nach und lässt für H4 (*p*_*H4*_ *=* *0,06*), H10 (*p*_*H10*_ *=* *0,06*) und H11 (*p*_*H11*_ *=* *0,08*) ein geringeres statistisches Signifikanzniveau (p_t‑test_ < 0,10) unterstellen. Diese Annahme bestätigt sich durch eine Berechnung von Pearsons Korrelationskoeffizient r, der eine starke positive Effektstärke *|r|* *>* *0,5* (Cohen 1992) für H2 (*|r*_*H2*_*|* *=* *0,71*), H4 (*|r*_*H4*_*|* *=* *0,60*), H10 (*|r*_*H10*_*|* *=* *0,59*) und H11 (*|r*_*H11*_*|* *=* *0,57*) nachweist. Obwohl für H12 (*p*_*H12*_ *=* *0,12, |r*_*H12*_*|* *=* *0,52*) eine entsprechende starke Effektstärke nachgewiesen werden kann, reicht das Signifikanzniveau (*p*_*H12*_ *=* *0,12*) nicht aus um eine ausreichende statistische Signifikanz zu unterstellen. Daraus ergibt sich die Annahme der Hypothesen H2, H4, H10 und H11 (siehe Tab. 4).

**Tab. 4:** Ergebnisse der statistischen Auswertung

Hypothese	Vergleichspaar	*p* (Shapiro Wilk)	*p* (gepaarter t‑test)	Effektstärke r
H1	MST1 – VR1	0,7494	1,0000	0,0000
**H2**	MST2 – VR2	0,3256	**0,0220**	**0,7068**
H3	MST3 – VR3	0,02545	0,5320	0,2246
**H4**	MST4 – VR4	0,2566	**0,0660**	**0,6019**
H5	MST5 – VR5	0,6246	1,0000	0,0000
H6	MST6 – VR6	0,5043	0,5250	0,2285
H7	MST7 – VR7	0,0811	0,4910	0,2473
H8	MST8 – VR8	0,3645	0,3470	0,3333
H9	MST9 – VR9	0,1704	0,1820	0,4594
**H10**	MST10 – VR10	0,4144	**0,0730**	**0,5895**
**H11**	MST11 – VR11	0,1604	**0,0820**	**0,5745**
H12	MST12 – VR12	0,01912	0,1220	0,5216

Im Folgenden werden insbesondere die angenommenen Hypothesen (H2, H4, H10 und H11) inhaltlich betrachtet und analysiert. Außerdem erörtern wir welche VR-Aspekte oder SECI-Modi von der Datenlage nicht abgedeckt werden. Tab. 5 zeigt eine Hypothesenübersicht, in welcher alle Hypothesen ausformuliert beschrieben sind und die angenommen Hypothesen H2, H4, H10 sowie H11 hervorgehoben sind.

**Tab. 5:** Hypothesenübersicht

Hypothese	Beschreibung
H1	Das Erleben bzw. Eintauchen in die virtuelle Umgebung („Immersion“) hat einen positiven Einfluss auf die SECI-Sozialisierung
**H2**	*Das Erleben bzw. Eintauchen in die virtuelle Umgebung („Immersion“) hat einen positiven Einfluss auf die SECI-Externalisierung*
H3	Das Erleben bzw. Eintauchen in die virtuelle Umgebung („Immersion“) hat einen positiven Einfluss auf die SECI-Kombination
**H4**	*Das Erleben bzw. Eintauchen in die virtuelle Umgebung („Immersion“) hat einen positiven Einfluss auf die SECI-Internalisierung*
H5	Die simulierte physische Präsenz der virtuellen Umgebung hat einen positiven Einfluss auf die SECI-Sozialisierung
H6	Die simulierte physische Präsenz der virtuellen Umgebung hat einen positiven Einfluss auf die SECI-Externalisierung
H7	Die simulierte physische Präsenz der virtuellen Umgebung hat einen positiven Einfluss auf die SECI-Kombination
H8	Die simulierte physische Präsenz der virtuellen Umgebung hat einen positiven Einfluss auf die SECI-Internalisierung
H9	Die Möglichkeiten der Interaktivität in der virtuellen Umgebung hat einen positiven Einfluss auf die SECI-Sozialisierung
**H10**	*Die Möglichkeiten der Interaktivität in der virtuellen Umgebung hat einen positiven Einfluss auf die SECI-Externalisierung*
**H11**	*Die Möglichkeiten der Interaktivität in der virtuellen Umgebung hat einen positiven Einfluss auf die SECI-Kombination*
H12	Die Möglichkeiten der Interaktivität in der virtuellen Umgebung hat einen positiven Einfluss auf die SECI-Internalisierung

Der stärkste nachgewiesene positive Effekt (H2) von *Immersion *(also dem Eintauchen in die virtuelle Welt) auf die *Externalisierung* von Wissen kann durch die erhöhte Greifbarkeit des Problemverständnisses sowie die informellen Interaktionsmöglichkeiten der virtuellen Welt begründet werden. Des Weiteren zeigt der nachgewiesene Effekt von *Immersion* auf die *Internalisierung* von Wissen (H4) den Zusammenhang zwischen dem Eintauchen in die virtuelle Welt und der Umwandlung des während des Versuches explizit Angewandten in implizites Wissen auf. Die *Interaktivität* hat gemäß H10 und H11 sowohl einen starken Effekt auf die *Externalisierung* als auch auf die *Kombination*. Insbesondere die bereits genannten nutzbaren und interaktive IFX-Elemente (3-dimensionale Texturobjekte wie z. B. Gegenstände, Architekturen oder Tiere (VR Expert 2020)), die während des Versuchs zur Interaktion und Anschaulichkeit bereitstanden, boten hier einen Mehrwert gegenüber MS Teams. Sowohl die *Externalisierung* und insbesondere die *Kombination* profitieren von der Möglichkeit Gegenstände und Sachverhalte mit virtuellen physischen Texturobjekten darzustellen, um vorhandenes explizites sowie neues Wissen zu sammeln, transformieren, verarbeiten und sortieren. Ein konkreter Rückschluss aus dieser Studie wäre zum Beispiel eine Empfehlung, für welche Arten von Wissensgenerierungs-Workshops die Technologie VR nennenswerte Vorteile bietet. Zum einen ist die Annahme von H2 und H10 ein Indikator dafür, VR insbesondere dann einzusetzen, wenn es darum geht aus impliziten Wissen explizite Ergebnisse zu generieren. Beispiele dafür wären sowohl Brainstorming, Prototyping als auch DT-Workshops. Die Annahmen von H4 bzw. H11 implizieren den Einsatz von VR für die Aufbereitung oder Transformation von expliziten Wissen (z. B. bei der Erstellung von Wissensdatenbanken bzw. -netzwerken) bzw. für die direkte Übertragung oder Umwandlung von explizitem in implizites Wissen (z. B. bei Schulungen in denen Regularien in Verhaltensweisen transformiert werden sollen).

Auffällig ist in jedem Fall, dass lediglich Hypothesen angenommen werden konnten, die die VR-Aspekte *Immersion* und *Interaktivität* als Ausgangspunkt für eine Verbesserung der jeweiligen Wissensgenerierungsmodi (zwei Mal *Externalisierung* sowie je einmal *Kombination* und *Internalisierung*) unterstellen. Eine mögliche Erklärung dafür bietet die kurze Zeitspanne in VR (<45 min), sowie die Tatsache, dass es sich bei den Teilnehmenden größtenteils um VR-Erstnutzende handelte, die bis auf die Einrichtung und ein erstes VR-Tutorial noch keine VR-Erfahrung hatten und sich daher noch nicht auf die situative Präsenz an einem virtuellen Ort einlassen konnten. Mindestens genauso erstaunlich ist die Tatsache, dass keine verbesserte *Sozialisierung* (Austausch von impliziten Wissen) nachgewiesen konnte, obwohl gemeinsame praktische Erfahrungen in der virtuellen Umgebung während der Versuchsdurchführung stattfinden konnten. Mögliche Erklärungen dafür bieten aufgetretene technische Schwierigkeiten beim Eintreten in den virtuellen Raum und der schnelle fachliche Einstieg in die Thematik der Fallstudie. Einen weiteren möglichen Störfaktor stellt auch die Tatsache dar, dass die Teilnehmenden mit MS Teams bestens vertraut waren, während die Vorerfahrungen mit VR eher gering waren.

Neben der quantitativen Auswertung, haben wir den Teilnehmenden im Nachgang auch einige offene, qualitative Fragen zu den Vor- bzw. Nachteilen der beiden eingesetzten Tools (MS Teams sowie EngageVR) gestellt. Die qualitativen Rückmeldungen reichten dabei von: „Gute Darstellbarkeit von Situationen und Problemen. [Das] interaktive Arbeiten regt […] mehr zur Kommunikation an, wodurch die Produktivität gesteigert wird“ bis: „Es ist viel Potenzial vorhanden, jedoch erfordert dies erst eine Einfindung in die Bedienung“. Positiv wurden dabei insbesondere das Präsenzgefühl, die gute Darstellbarkeit von Situationen und Problemen sowie die Kreativitätsanregung in EngageVR bezeichnet. Im Gegensatz dazu punktet MS Teams durch die leichtere Bedienung. Gerade die Bedienung von Whiteboards stellte sich in VR Ungeübten als Herausforderung dar. Darüber hinaus wurde die sogenannte „Motion-Sickness“, ein Schwindel-Gefühl aufgrund des Widerspruches von künstlicher Bewegung in VR ohne physische Bewegung (Bezmalinovic 2021), als ein Störfaktor genannt.

## Diskussion und Forschungsausblick

Generell stellt die technologische Entwicklung hin zu sogenannten „eXtended Realities“, zu denen auch die VR-Technologie (Milgram und Kishino 1994; Wohlgenannt et al. 2020) gehört, einen Paradigmenwechsel dar. Nicht umsonst hat Facebook im Jahr 2014 den VR-Spezialisten Oculus VR für zwei Milliarden Dollar übernommen, mit der Vision, eine völlig neue Kommunikationsplattform zu schaffen – ähnlich den heutigen Smartphones (Lindner 2014). Zwar gibt es noch Hürden auf dem Weg zum neuen Medium der Virtual-Reality-Plattform (z. B. Motion Sickness oder Hardware-Verfügbarkeit), aber aussichtsreiche VR-Geschäftsmodelle entstehen bereits abseits der Spieleindustrie (z. B. dpa 2021). Das Potenzial, die typische Arbeitsweise, wie wir sie heute kennen, zu verändern, ist deutlich erkennbar.

Unsere Studie hatte das Ziel die Diskrepanz zwischen praktischer Anwendung und theoretischer Forschung hinsichtlich VR zu verringern. Die Gegenüberstellung der Anwendungsszenarien von VR gegenüber konventionellen Tools unter Berücksichtigung von SECI-Phasen und DT-Prinzipien trägt dazu bei konkrete Einsatzgebiete für den gewinnbringenden Einsatz von VR in der Wissensgenerierung zu identifizieren. Unsere grundlegende Annahme, dass VR die Wissensgenerierung im Allgemeinen verstärken kann, sehen wir trotz der begrenzten Aussagekraft durch den geringen Stichprobenumfang bestärkt. Nichtsdestotrotz sollten künftige Versuchsaufbauten in größerem Umfang unter Realbedingungen mit mehr Teilnehmenden und längeren Versuchsdauern geplant und durchgeführt werden, um die Aussagekraft der Ergebnisse zu erhöhen. Darüber hinaus sollten bei künftigen Untersuchungen unserer Einschätzung nach zusätzlich weiche Faktoren wie z. B. Blickkontakt und die Fragen, ob sich die Teams vor den VR-Meetings schon kannten oder ob Zeit für Smalltalk vorab eingeräumt wurde, mehr Berücksichtigung finden.

Aus unseren Ergebnissen lassen sich einige Hinweise ableiten, unter welchen Bedingungen sich z. B. DT in der Wissensgenerierung in der betrieblichen Praxis gewinnbringen einsetzten lassen kann. Beispielsweise zeigt die Annahme von H2 und H10, dass die SECI-Phase *Externalisierung *großes Potenzial für VR bietet. Daraus lässt sich schließen, dass VR insbesondere da eingesetzt werden sollte, wo ein Transfer von impliziten in explizites Wissen stattfindet, also dort, wo Wissen erfasst und transferiert wird (z. B. also in VR-Trainings). Darüber hinaus zeigen unsere Daten, dass die VR-Aspekte *Immersion *(H2, H4) und *Interaktivität* (H10, H11) in Bezug auf Wissensgenerierung relevanter sind als *Präsenz*. Konkret bedeutet das, dass der Fokus bei VR-Umgebungen zur Wissensgenerierung eher darauf liegen sollte, diese immersiver und interaktiver zu gestalten, anstatt das Raumgefühl als Präsenzfaktor zu verstärken. Dafür könnten beispielsweise weitere Interaktionsmöglichkeiten (z. B. Laserpointer für Präsentationen) oder weitere Sinneseindrücke (z. B. haptisches Feedback) miteinbezogen werden. Aufbauend auf diesen Ergebnissen können konkrete Anwendungen entwickelt und entsprechend getestet werden. Basierend auf unseren Ergebnissen wäre daher beispielsweise der Vergleich von Onboarding-Prozessen in informellen Umgebung zwischen herkömmlichen Tools und VR geeignet, um aus unseren eher theoretischen Ergebnissen konkretere praktische Hinweise ableiten zu können.

VR stellt eine potenzielle Disruption sowohl im Arbeits- als auch im Sozialleben dar. Die Anwendung in der betrieblichen Praxis, wie sie in dieser Studie erforscht wurde, ist insbesondere durch Skepsis gegenüber neuen Technologien, unausgereifte und teure Hard- und Software sowie fehlende anwendungsorientierte Forschung begrenzt. Dennoch können weitere Studien dieser Art mit spezifischen Fragestellungen aus den verschiedensten Forschungsbereichen dem Mangel an konkreten Anwendungsszenarien für VR in der betrieblichen Praxis entgegenwirken.
